# Phenotypic Modulation of Corpus Cavernosum Smooth Muscle Cells in a Rat Model of Cavernous Neurectomy

**DOI:** 10.1371/journal.pone.0105186

**Published:** 2014-08-15

**Authors:** Fan Yang, Jian F. Zhao, Qi Y. Shou, Xiao J. Huang, Gang Chen, Ke B. Yang, Shi G. Zhang, Bo D. Lv, Hui Y. Fu

**Affiliations:** 1 Department of Urology, The Second Affiliated Hospital, Zhejiang Chinese Medical University, Hangzhou, China; 2 Laboratory Animal Research Center, Zhejiang Chinese Medical University, Hangzhou, China; 3 Central Laboratory, The Second Clinical Medical College, Zhejiang Chinese Medical University, Hangzhou, China; 4 Andrology Laboratory on Integration of Chinese and Western Medicine, Zhejiang provincial Key Laboratory of Traditional Chinese Medicine, Hangzhou, China; University College London, United Kingdom

## Abstract

**Background:**

Patients undergoing radical prostatectomy (RP) are at high risk for erectile dysfunction (ED) due to potential cavernous nerve (CN) damage during surgery. Penile hypoxia after RP is thought to significantly contribute to ED pathogenesis.

**Aim:**

We previously showed that corpora cavernosum smooth muscle cells (CCSMCs) undergo phenotypic modulation under hypoxic conditions *in vitro*. Here, we studied such changes in an *in vivo* post-RP ED model by investigating CCSMCs in bilateral cavernous neurectomy (BCN) rats.

**Methods:**

Sprague-Dawley rats underwent sham (n = 12) or BCN (n = 12) surgery. After 12 weeks, they were injected with apomorphine to determine erectile function. The penile tissues were harvested and assessed for fibrosis using Masson trichrome staining and for molecular markers of phenotypic modulation using immunohistochemistry and western blotting. CCSMC morphological structure was evaluated by hematoxylin-eosin (H&E) staining and transmission electron microscopy (TEM).

**Results:**

Erectile function was significantly lower in BCN rats than in sham rats. BCN increased hypoxia-inducible factor-1α and collagen protein expression in corpora cavernous tissue. H&E staining and TEM showed that CCSMCs in BCN rats underwent hypertrophy and showed rough endoplasmic reticulum formation. The expression of CCSMC phenotypic markers, such as smooth muscle α-actin, smooth muscle myosin heavy chain, and desmin, was markedly lower, whereas vimentin protein expression was significantly higher in BCN rats than in control rats.

**Conclusions:**

CCSMCs undergo phenotype modulation in rats with cavernous neurectomy. The results have unveiled physiological transformations that occur at the cellular and molecular levels and have helped characterize CN injury–induced ED.

## Introduction

Radical prostatectomy (RP) is considered to be the most effective therapy for patients with early-stage prostate cancer, with approximately 25% of them undergoing RP [1]. Due to the potential risk of damage to the cavernous nerves (CNs), erectile dysfunction (ED) is a highly prevalent complication of RP surgery [2] and significantly impairs the man's self-esteem and quality of life. Hypoxia is thought to be an etiological factor of RP-induced ED [3]-[5]. Provoked or spontaneous nocturnal erections play a critical role in the maintenance of male sexual health through reoxygenation of the corpus cavernosa [6]. Furthermore, at 12–15 weeks after CN injury, the collagen to smooth muscle ratio increases and this is accompanied by overexpression of the hypoxia inducible factor-1α (HIF-1α), an important transcription factor that responds to changes in oxygen pressure in the cellular environment [7], [8]. The exact mechanism underlying these changes is not well understood yet.

A better understanding of corpora cavernosum smooth muscle cell (CCSMC) function and its phenotypic transformations can help illuminate the pathogenesis of ED after RP. Unlike the myocardium and skeletal muscle cells which are terminally differentiated, vascular smooth muscle cells (VSMCs) maintain plasticity in cellular phenotype and can change from a contractile (differentiated) state to a synthetic (dedifferentiated) state in response to extracellular cues [9]. The synthetic state is characterized by a high level of proliferation, migration, extracellular matrix production, and vimentin overexpression and low-level expression of contractile cytoskeletal proteins such as smooth muscle (SM) α-actin (α-SMA), SM myosin heavy chain (SMMHC), and desmin [10]. Recently emerging evidence has shown that the phenotypic modulation of SM cells (SMCs) plays an important role in the pathogenesis of various diseases of the cardiovascular and respiratory systems, such as atherosclerosis, hypertension, and asthma among others [11]-[13]. However, to our knowledge, changes in CCSMCs in post-neurectomy rats have not yet been reported.

The present study was designed to evaluate the phenotypic alterations in CCSMCs of penile tissue in an *in vivo* rat model of post-RP ED.

## Materials and Methods

### Animals

Adult male Sprague-Dawley rats (SLRC Laboratory Animals, Shanghai, China) weighing 275–325 g were used in the experiments. The rats were raised using a 12∶12 light cycle at 24±1°C. The rats had free access to food and drinking water. They were separated into two groups: sham-operated (sham, n = 12) group and bilateral cavernous neurectomy (BCN, n = 12) group. All animals were handled in strict accordance with the recommendations in the ARRIVE guidelines [14]. The protocol was approved by the Committee on the Ethics of Animal Experiments of the University of Zhejiang Chinese medical university. All surgery was performed under sodium pentobarbital anesthesia, and all efforts were made to minimize suffering. Animals were sacrificed by an anaesthetic overdose intraperitoneal administration of sodium pentobarbital and then cervical dislocation was applied to rats for euthanasia.

### Establishment of a BCN Rat Model

The BCN procedures were performed according to methods described in a previous report [15]. All the rats were anaesthetized with an intraperitoneal injection of 3% sodium pentobarbital (0.1 ml/100 g); supplementary doses of sodium pentobarbital were used, when needed, to maintain anesthesia. A hypogastrium midline incision was made from the symphysis pubis to the mid-abdomen region. The dorsal lobes of the prostate were exposed, and then the CNs were identified and dissected and 5-mm segments were removed on both sides. For the sham-operated group, the CNs were dissected but not cut. Antibiotics were fed orally to all rats for 3 days after the operation.

### Measurement of Erectile Responses

A penile erection experiment was performed using the methods described in a previous report [16]. All lights (except some indirect light for observation in the quiet room) were turned off, after which the rats were kept in a transparent box for approximately 10 min so that they could adapt to the new surroundings. Then, they were subcutaneously injected with apomorphine (100 µg/kg; Sigma Chemical Company, St. Louis, Mo, USA), dissolved in normal saline solution, in the loose skin at the back of the neck. The status of penile erection was observed and recorded by two individuals for 30 min after the injection. Penis erections were identified with glans engorgement and appearance of the penile shaft. Erection rate was measured as the ratio of the number of rats that showed erections to the total number of rats.

### Staining and Immunohistochemistry

Penises were excised and then placed in 4% neutral buffered formalin overnight at 4°C and subsequently processed, embedded in paraffin, and sectioned at 4 µm. Hematoxylin-eosin and Masson trichrome staining was performed. The slides were photographed using a Nikon Eclipse 80i microscope (Nikon, Tokyo, Japan). Five non-overlapping images of Masson trichrome staining were captured from each slide at 400× magnification and semi-quantitative image analysis of Masson trichrome staining was performed as described previously using the Image-Pro Plus 6.0 software [17].

Sections were deparaffinized and rehydrated, and then retrieved with heat-induced epitope retrieval. Endogenous peroxidase was inhibited with 3% hydrogen peroxide (H_2_O_2_) and nonspecific antigen was blocked with 5% bovine serum albumin (BSA; Amresco, Solon, OH, USA). The slides were then incubated with the primary antibody (α-SMA [1∶100], HIF-1α [1∶100], and vimentin [1∶250], all from Abcam, Cambridge, UK) overnight at 4°C, rinsed 3 times in phosphate-buffered saline (PBS) for 5 min at room temperature, and incubated with a biotinylated secondary antibody (diluted 1∶100); this was followed by incubation with the streptavidin–biotin peroxidase complex (diluted 1∶100). Immunohistochemical detection was performed with 3,3′-diaminobenzidine tetrahydrochloride (DAB) following the manufacturer's instructions. Tissue sections were viewed with a Nikon Eclipse 80i microscope (Nikon, Tokyo, Japan) equipped with a camera. Images were captured using the NIS-Element S.F. 2.30 software at 40×, 100×, and 400× magnification. Five non-overlapping images were captured from each slide at 400× magnification. Semiquantitative image analysis was performed with the Image-Pro Plus 6.0 software, as described in a previous study [18].

### Transmission Electron Microscopy

The penile tissues were prepared as approximately 2 mm-thick-sections and fixed in 2.5% glutaraldehyde, and were further fixed in 1% osmium tetroxide and dehydrated in graded concentrations of ethanol; subsequently, they were infiltrated in graded resins and finally embedded in Epon epoxy resin. Ultrathin sections were cut with a diamond knife and stained with 5% uranyl acetate and lead citrate. Images were acquired by a fully trained expert by using a Tecnai 10 transmission electron microscope (Philips, Amsterdam, The Netherlands).

### Western blot analysis

Penises were excised, the urethra and tunica albuginea were removed, and the corpus cavernosum tissue samples were lysed in lysis buffer (20 mM Tris [pH 7.5], 150 mM NaCl, 1 mM Na_2_EDTA, 1 mM EGTA, 1% Triton X-100, 2.5 mM sodium pyrophosphate, 1 mM beta-glycerophosphate, 1 mM Na_3_VO_4_, and 1 µg/mL leupeptin) on ice and centrifuged at 12,000 rpm for 10 min at 4°C. The protein concentration was tested using a BCA protein assay kit (Pierce, Rockford, IL, USA). Forty micrograms of protein was loaded on 6–12% SDS-PAGE gels and transferred to polyvinylidene difluoride (PVDF) membranes (Bio-Rad). The membranes were treated with 5% BSA and then incubated overnight at 4°C with primary antibodies against HIF-1α, α-SMA, SMMHC, collagen-I, vimentin, and desmin: HIF-1α, 1∶3000; α-SMA, 1∶5000; SMMHC, 1∶1000; collagen-I, 1∶5000; vimentin, 1∶5000; and desmin, 1∶500. Desmin was purchased from Santa Cruz Biotechnology (Santa Cruz, CA) and the rest were from Abcam (Cambridge, MA, USA). Then, the membranes were washed and incubated with IRDye800 goat anti-rabbit or anti-mouse IgG secondary antibody (Li-Cor, Lincoln, NE) for 2 h. Immunoreactive proteins were visualized with the Odyssey Infrared Imaging System (Li-Cor).

### Statistical Analysis

All experiments were performed in triplicates. Data were analyzed using one-way ANOVA followed by Student's t-test using the SPSS 15.0 software. Data have been expressed as mean ± SEM. *P*<0.05 was considered to indicate statistical significance.

## Results

### Assessment of erectile response

The erectile response in the BCN group was significantly lower than that in the sham group (Table 1). After being injected with apomorphine, the rats in the BCN group exhibited a lower erection rate than the sham-operation group (*P*<0.05); no rats in the BCN group showed any erection within 30 min, while 11 rats showed a clear erection and 1 rat showed a slight erection in the sham group.

**Table 1:** Penile erectile response to injection of apomorphine (100 µg/kg).

Group	Reaction	Non-reaction	Total	Erectile rate (%)
Sham	11	1	12	91.67
BCN	0	12	12	0*
Total	11	13	24	55

Data were analyzed by chi-square test (x_c_^2^ = 12.93). *P<0.05. BCN group, n = 12; sham-operation group, n = 12. BCN = bilateral cavernous neurectomy; Sham = sham operation.

### Assessment of HIF-1α expression

**Figure 1:**
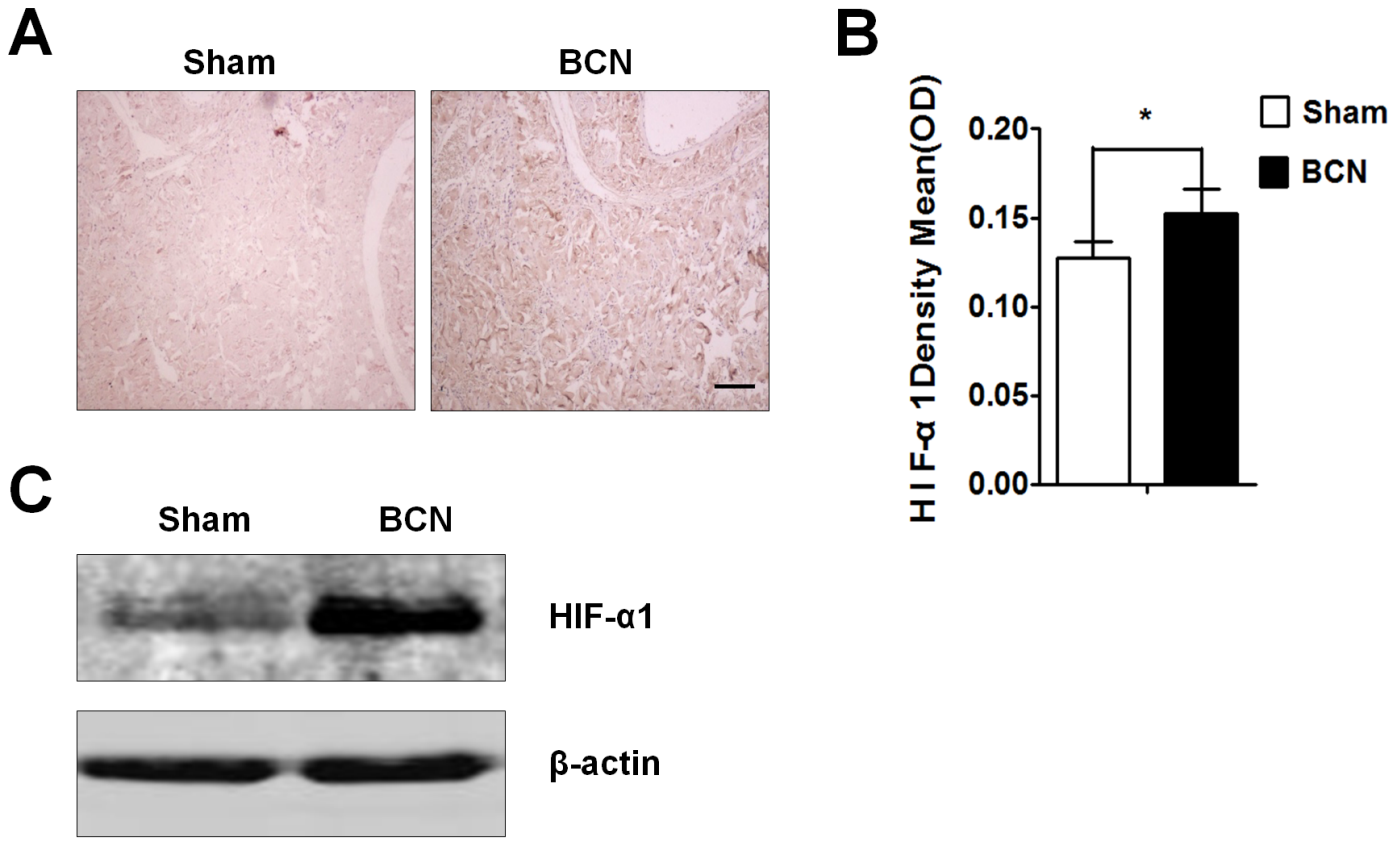
Expression of HIF-1α in the corpus cavernosum tissues of BCN rats. (**A**) Rats were killed at 12 weeks after BCN, and penis samples were prepared for detection of HIF-1α expression using immunohistochemical staining (100×, Scale bars = 200 µm). (**B**) Semi-quantitative image analysis of HIF-1α expression in corpus cavernosum tissues was performed using the Image-Pro Plus 6.0 software. *P<0.05 compared with the sham rats (n = 5/group). (**C**) Rat corpus cavernosum tissues were harvested and its lysates were used for western blotting with primary antibodies against α-SMA, Vim, SMMHC, desmin, and β-actin (Sham group: n = 3, BCN group: n = 4). All results are representative of three independent experiments. BCN = bilateral cavernous neurectomy; Sham = sham operation.

The HIF-1α protein expression in penile tissue from the sham and BCN groups was evaluated by immunohistochemistry. The results showed that, at 12 weeks after surgery, very few areas in the tissues from the sham group were positive for HIF-1α protein expression, whereas tissues from the BCN group exhibited strong expression in many areas (Figure 1A). Total HIF-1α levels were markedly higher in penile tissues of the BCN group than in those of the sham group (*P*<0.05; Figure 1B). Western blot results also showed increased expression of HIF-1α in BCN rats (Figure 1C).

### Assessment of collagen fiber expression

**Figure 2:**
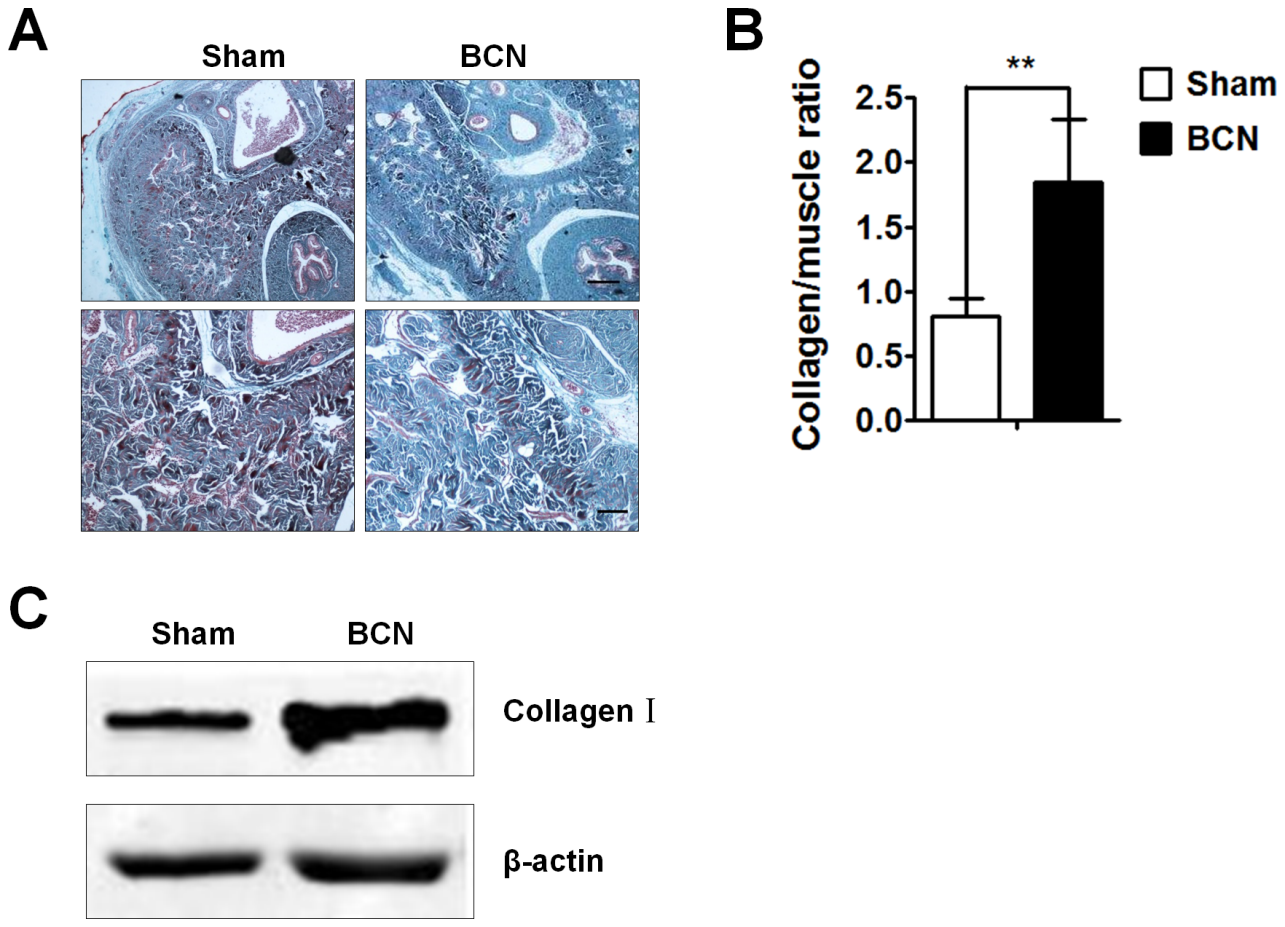
Analysis of corpus cavernosum tissue fibrosis in BCN rats. (**A**) Penis samples were prepared for the detection of corpus cavernosum tissue fibrosis using Masson trichrome staining (Upper-line: 40×, Scale bars = 500 µm, down-line: 100×, Scale bars = 200 µm). (**B**) Semi-quantitative image analysis of collagen/muscle ratio in corpus cavernosum tissues was performed using the Image-Pro Plus 6.0 software. **P<0.01 compared with the sham rats (n = 5/group). (**C**) Western blot analyses measuring collagen-I protein expression levels in penile tissues of both groups (Sham group: n = 3, BCN group: n = 4). All results are representative of three independent experiments. BCN = bilateral cavernous neurectomy; Sham = sham operation.

The extent of corpus cavernosum fibrosis was evaluated by Masson trichrome staining of the penile tissue from the sham and BCN groups. The degree of penile fibrosis in BCN rats was more severe than that in the sham group; photomicrographs from a typical experiment are showed in Figure 2A. Computer-assisted analysis indicated that the collagen/muscle ratio was 0.80±0.14 in sham group rats. BCN significantly increased this ratio (1.84±0.49, *P*<0.01, Figure 2B). Western blot results also showed a remarkable increase in the expression of collagen-I in BCN rats (Figure 2C).

### Assessment of CCSMC morphological features

**Figure 3:**
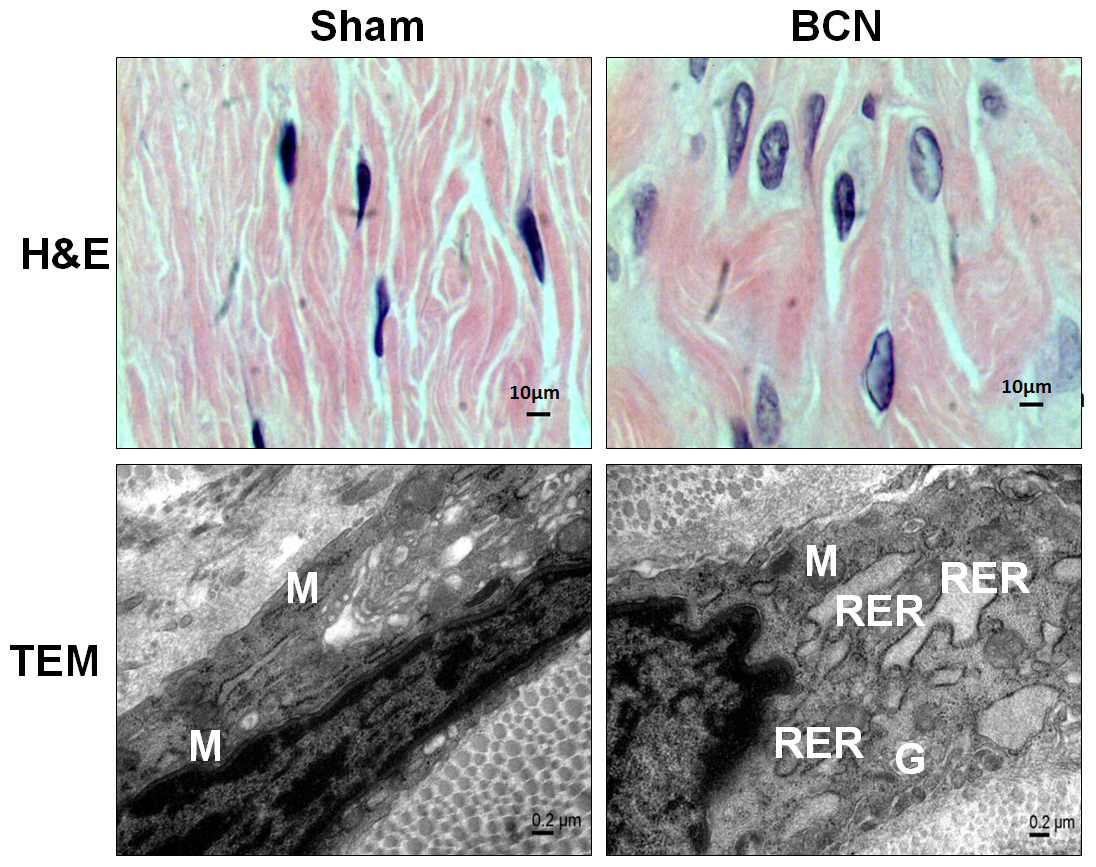
Observation of the morphology and structure of corpus cavernosum smooth muscle cells in BCN rats. Representative H&E photomicrograph (1000×, Scale bars = 10 µm) and transmission electron microscopy photomicrographs (40,000×, Scale bars = 0.2 µm) of CCSMCs. The protocol used is described in the methods section. M: mitochondria; RER: rough endoplasmic reticulum; G: Golgi apparatus. BCN = bilateral cavernous neurectomy; Sham = sham operation.

Prolonged hypoxia for 12 weeks resulted in significant cellular hypertrophy. H&E staining images were acquired under high magnification (1000×), using an oil immersion technique to aid visualization. Compared with the CCSMCs from the sham group, those from the BCN group showed an increased cell diameter, suggesting hypertrophy (Figure 3A). We also observed ultrastructural changes along with changes in cellular morphological features that included high levels of myofilament loss and rough endoplasmic reticulum (RER) formation (Figure 3B).

#### Assessment of proteins associated with the CCSMC phenotype

**Figure 4:**
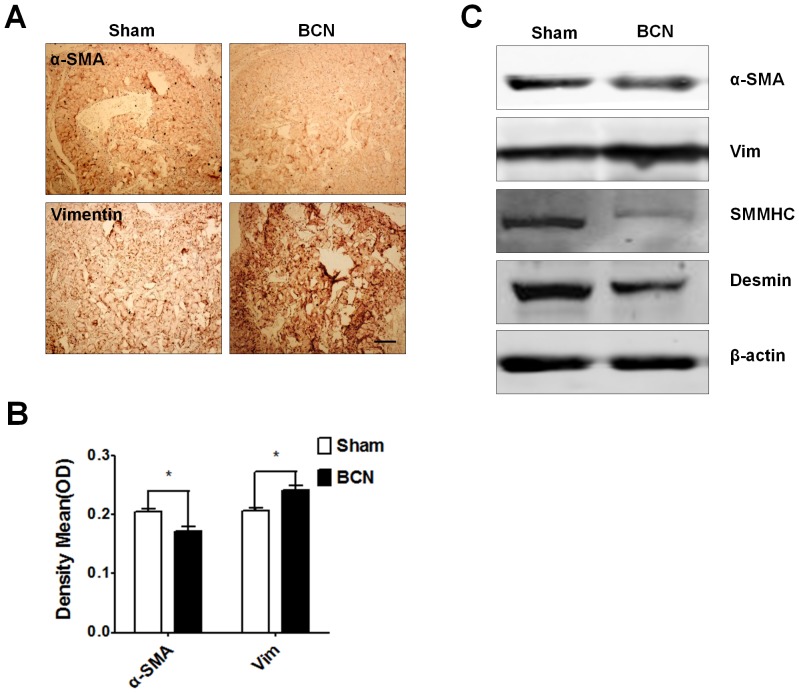
Measurement of the phenotypic modulation of CCSMCs in BCN rats. (**A**) Representative immunohistochemical staining photomicrographs for smooth muscle α-actin (α-SMA) and vimentin proteins (100×, Scale bars = 200 µm). (**B**) Semi-quantitative image analysis of α-SMA and vimentin expression in both groups. *P<0.05 compared to the sham rats (n = 5/group). (**C**) Western blot analysis of the levels of α-SMA, vimentin, desmin, SM myosin heavy chain (MHC), and β-actin proteins in penile tissue of both groups (Sham group: n = 3, BCN group: n = 4). All results are representative of three independent experiments. BCN = bilateral cavernous neurectomy; Sham = sham operation.

The expression of phenotypic protein markers was evaluated by immunohistochemical staining of penile tissue from the sham and BCN groups. Photomicrographs from a typical experiment are shown in Figure 4A. Computer-assisted analysis of these images indicated that α-SMA expression had significantly decreased and vimentin expression had dramatically increased in the BCN rats as compared to that in the sham group (P<0.05, Figure 4B). Using western blot analyses, we found that the expression of α-SMA, SMMHC, and desmin was downregulated, whereas the expression of vimentin was upregulated in the BCN rats (Figure 4C).

## Discussion

To our knowledge, our study is the first to demonstrate that CCSMCs undergo phenotypic modulation in BCN rats. BCN was also shown to cause a significant decrease in erectile function in these rats. HIF-1α and collagen proteins were highly expressed in the penile corpora cavernous tissues of rats following BCN exposure. Additionally, SMC contractile proteins of the corpora cavernosum, such as α-SMA, SMMHC, and desmin, were downregulated in BCN rats, whereas synthesis of the SMC phenotypic marker protein vimentin was significantly upregulated.

Increase in collagen synthesis and in hypoxia inducible factor-1α (HIF-1α) and transforming growth factor-β1 (TGF-β1) expression has been observed after cavernous neurectomy. [7] Our results confirm the direct relationship that has been hypothesized to exist between hypoxia and corpus cavernosum fibrosis in penile tissue after neurectomy. It is widely believed that the lack of sleep-related erectile episodes, induced by BCN, is one of the most important reasons for hypoxia in the corpus cavernosum [19] suggesting that improvements in cavernous blood flow and reoxygenation of the penis provide effective therapies for preserving the erectile function after RP.

CCSMCs are known to be the most important cells in the male erection process [20]. It has been observed that the abnormalities in CCSMCs that are attributed to CN injury reduce the ability of the tissue to establish sufficient intracavernous pressure (ICP) for blocking the veins that traverse under the tunica albuginea and egress from the corporal bodies.[21] SMCs exist in contractile and synthetic phenotypic states, even in mature organs. SMCs maintain high plasticity and undergo phenotypic modulation in response to local cellular stimuli, such as hypoxic conditions [22].

Cellular morphological features are an important indicator of SMC behavior [23]. In culture, a single cell in the contractile state exhibits a spindle or rhomboid shape and contains a large number of rich myofilaments [24]. SMCs with a synthetic phenotype appear broader with a bigger diameter and show ECM deposition and formation of RER [25]. In this study, the CCSMCs in BCN rat penile tissues displayed similar changes in their morphology (increased cell diameter) and structure (increased loss of myofilaments and increased RER formation).

A variety of SMC-specific target gene and gene products have been identified as useful markers of the phenotypic state of the SMCs. These include a large number of contractile proteins, including α-SMA, SMMHC, and desmin. α-SMA was the first protein found to be expressed in the contractile state of the SMC in mature organs and serves as a reliable marker [11]. SMMHC is highly restricted to SMCs and therefore serves as an ideal SMC marker. Both desmin and vimentin are cytoskeletal proteins of the intermediate filament. Desmin is believed to be another contractile SMC marker [26], while high expression of vimentin in impaired vascular tissue has been used as a marker for synthetic SMC [27]. These four proteins (α-SMA, SMMHC, desmin, and vimentin) have been used in this study to characterize SMC phenotypes. Our results showed that CCSMCs subject to BCN-induced hypoxic conditions displayed changes in protein expression, that is, decreased expression of α-SMA, SMMHC, and desmin and increased expression of vimentin, that were consistent with other published data on phenotype modulation in SMCs.

## Conclusions

Our results demonstrated that CCSMCs undergo a shift in phenotypes from a contractile state to a synthetic state in a rat model of BCN. This phenotypic modulation could play a key role in the pathogenesis of post-RP ED. The exact molecular mechanism underlying this effect remains to be further clarified.
